# A Review of Self-Compassion as an Active Ingredient in the Prevention and Treatment of Anxiety and Depression in Young People

**DOI:** 10.1007/s10488-021-01170-2

**Published:** 2021-09-24

**Authors:** Sarah J. Egan, Clare S. Rees, Joanna Delalande, Danyelle Greene, Grace Fitzallen, Samantha Brown, Marianne Webb, Amy Finlay-Jones

**Affiliations:** 1 enAble Institute, Faculty of Health Sciences, Curtin University, GPO Box U1987, Perth, 6845 Australia; 2 Discipline of Psychology, School of Population Health, Curtin University, GPO Box U1987, Perth, 6845 Australia; 3 Telethon Kids Institute, Perth Children’s Hospital, 15 Hospital Ave, Nedlands, Perth, WA 6009 Australia

**Keywords:** Self-compassion, Adolescents, Youth, Anxiety, Depression, Review

## Abstract

Previous meta-analyses have found higher self-compassion is associated with lower anxiety and depression. The aim of this study was to investigate the efficacy of self-compassion as an active ingredient in the treatment and prevention of anxiety and depression in youth. This was conducted through (i) a systematic review of the literature and (ii) qualitative consultation with young people and researchers in self-compassion. Fifty studies met our inclusion criteria. Eight studies evaluated self-compassion interventions among youth aged 14–24, and the remaining studies measured the association between self-compassion and anxiety, and/or depression among this age group. Qualitative interviews were conducted with four self-compassion researchers. Interviews were also conducted in two rounds of consultation with 20 young people (*M* age = 18.85 years, age range 14–24 years). Higher self-compassion was related to lower symptoms of anxiety, *r* =  − 0.49, 95% CI (− 0.57, − 0.42), and depression, *r* =  − 0.50, 95% CI (− 0.53, − 0.47). There was evidence for self-compassion interventions in decreasing anxiety and depression in young people. Consultation with young people indicated they were interested in self-compassion interventions; however, treatment should be available in a range of formats and tailored to address diversity. Self-compassion experts emphasised the importance of decreasing self-criticism as a reason why self-compassion interventions work. The importance of targeting self-criticism is supported by the preferences of young people who said they would be more likely to engage in a treatment reducing self-criticism than increasing self-kindness. Future research is required to add to the emerging evidence for self-compassion interventions decreasing symptoms of anxiety and depression in young people.

**Supplementary Information:**

The online version contains supplementary material available at 10.1007/s10488-021-01170-2.

## Introduction

There are two main approaches that underpin the study of self-compassion. Neff (2003a) proposes that self-compassion involves taking a balanced and self-supportive approach in times of difficulty and remembering one is not alone in one’s suffering. Gilbert (2017) states that compassion involves courage to engage in suffering, and wisdom on how best to act. Gilbert (2017) argues that self-criticism, defined as self-critical thoughts towards oneself (e.g., “I’m not good enough”), is a central process targeted by compassion interventions. The Self-Compassion Scale (SCS; Neff, 2003a) is the most commonly used measure of self-compassion consisting of six subscales, three representing positive factors; self-kindness, common humanity, and mindfulness and three negative factors; self-judgement, isolation, and over-identification. While many studies demonstrating a link between higher self-compassion and lower symptoms of anxiety/depression have used the SCS (Neff, 2003a), it has been argued that incorporating the negative subscales is problematic as items overlap with psychological symptoms, which over-inflates the relationship of the total self-compassion score with anxiety/depression (see Muris & Otgaar, 2020 and Neff, 2020 for a rebuttal). Further, some authors have argued self-compassion and self-kindness are distinct constructs (e.g., Gilbert et al., 2019).

Self-compassion is hypothesised to be an “active ingredient” in prevention and intervention for anxiety and depression in young people. Active ingredients refer to core aspects of psychological interventions that are effective in the prevention and treatment of anxiety and depression in young people (Wellcome Trust, 2021). Despite variation in the definition of self-compassion, it is proposed as an active ingredient for symptoms of anxiety and depression in young people on the basis of Marsh et al.’s (2018) findings in adolescents of a large effect size between higher self-compassion and lower psychological distress (including anxiety, depression and stress; *r* =  − 0.55). Further, there is evidence for the efficacy of self-compassion interventions in reducing symptoms of anxiety and depression in youth. Some of these interventions are based on Gilbert’s Compassion Focused Therapy (CFT) and primarily focus on cognitive therapy techniques (e.g., Arimitsu, 2016; Foxx et al., 2020) while Mindful Self-Compassion (MSC) interventions are more closely aligned to Neff’s definition (e.g., Bluth et al., 2015, 2016). Previous meta-analyses have demonstrated the efficacy of self-compassion interventions (including CFT and MSC) for anxiety and depression, however none have specifically examined their efficacy among young people. Ferrari et al. (2019) conducted the most recent meta-analysis and found moderate effects of interventions on anxiety (*g* = 0.57) and depression (*g* = 0.66). However, they did not disaggregate by age, and they did not find any studies specifically with children and adolescents and therefore could not answer how self-compassion may be an active ingredient of interventions for anxiety and depression in young people. Further, Ferrari et al. (2019) only examined randomised controlled trials (RCTs) and did not report in detail on intervention content, which is critical for providing insight into for whom, and under which circumstances, self-compassion interventions work.

The aim was to synthesise the evidence and provide expert input from young people and researchers on self-compassion as an active ingredient of interventions for young people (aged 14–24) at risk of, or currently experiencing, symptoms of anxiety and depression. The overarching research question was: *“drawing inferences from the current evidence: in which ways and in which contexts and for whom does your…active ingredient appear to work, and why; and in which ways and in which contexts and for whom does it appear not to work, and why?”* (Wellcome Trust, 2020). While there have been meta-analyses examining self-compassion, anxiety and depression in adolescents (Marsh et al., 2018) and the efficacy of self-compassion interventions (Ferrari et al., 2019) there are no reviews integrating evidence for self-compassion in young people or that integrate the perspectives of young people. Young people aged 14–24 years were included given evidence for peak onset of psychological disorders during this period (Jones, 2018). Quantitative studies were examined in a systematic review, and where applicable, a meta-analysis, in addition to consultation with young people and researchers.

## Methods

### Search Strategy and Selection Criteria

The literature search using the search terms and in Table 1, was conducted on 15 June 2020 in Embase, PubMed/Medline, PsycINFO and Web of Science. Inclusion criteria were (a) in English; (b) used self-compassion or self-kindness for a significant part of an intervention, defined as at least 75% or more of the sessions containing a focus on self-compassion as judged by the senior authors who are experienced clinicians in self-compassion interventions (SE and CR), (c) measured self-compassion as well as symptoms of depression and/or anxiety (including related symptoms e.g., worry, trauma); (d) included participants aged range between 14 and 24 years (or if age range not available through the journal article and author contact, reported a mean age between 14 and 24); and (e) published between 1 January 1980 and 15 June 2020. The restriction of 1980 onwards was chosen due to limited self-compassion research prior to this year, as evidenced by only 18 search results prior to this year, with none of these studies measuring self-compassion. For intervention studies, there were no limits regarding timepoints for measurement, i.e., studies could include either post treatment or both pre-post treatment measurements. The exclusion criterion was single case designs. Grey literature and desk-drawer studies were also sought through emails sent to self-compassion researchers for in-preparation manuscripts. The inclusion criteria were applied to title and abstracts in the initial screening stage to remove duplicates and papers that were clearly out of range, for example not a study of self-compassion by MW). After this initial screening stage, full text screening was applied to all papers by MW.

**Table 1:** Search terms and selection criteria

Compassion	Psychopathology/Wellbeing	MeSH Terms
Self-compassion	Anxiety	Anxiety disorders
Compassionate mind	Generalised anxiety disorder	Depression
Compassion	Depression	Mental health
Self-kindness	Mental health	Panic disorder
Self-soothing	Emotion regulation	Emotional regulation
Loving-kindness	Specific phobia	Psychological distress
Self-warmth	Panic	Obsessive–compulsive disorder

### Procedure

The PRISMA statement (Page et al., 2021) was followed and the protocol registered on PROSPERO in June 2020 (CRD42020188990). Article screening was conducted by MW, and a random 30% by DG, who had near perfect agreement (96%: Cohen’s Kappa = 0.90, *p* < 0.001). Discrepancies were resolved by SE and CR. The determination of whether an intervention was self-compassion was critical in the screening and selection of papers. All papers that were potentially relevant even if they did not explicitly label the intervention as self-compassion were carefully reviewed in terms of content by the senior authors who are experts in self-compassion. This involved reviewing journal article text and tables of session content for the intervention to determine through this clinical and research expertise in self-compassion that the intervention was indeed a self-compassion intervention. Where available through author contact, full treatment manuals for the interventions were also examined to determine the treatment content. The criteria of the intervention containing 75% content which was a self-compassion intervention was coded as “yes” or “no” to the 75% criteria. There was 100% agreement between the two senior authors (SE and CR) and no discrepancies, therefore inter-rater reliability in these ratings were not calculated.

A meta-analysis was conducted for associations between self-compassion and both anxiety and depression (i.e., question 2) in JASP (JASP Team, 2020). Hedges random effects model was used to pool the correlation coefficients across-studies (Hedges & Olkin, 1985). Study quality, as assessed by the National Institutes of Health (2014) Quality Assessment tools, measure of anxiety/depression and country were assessed as moderators in a meta-regression for both associations. Measure of anxiety/depression was chosen as a moderator because the method of measuring anxiety/depression differed across the studies (e.g., trait, state, social anxiety). Country was chosen as a moderator because culture and other location-based norms might impact the associations between self-compassion and both anxiety and depression (e.g., Neff et al., 2008). All moderators were dummy coded to contrast effect sizes across different groups of studies. Study quality was coded as follows, 1 = low (reference category), 2 = fair, and 3 = high. For measure of anxiety/depression and country a reference category had to be determined. We chose the reference category to be the category with the largest number of effect sizes. This was determined to be the STAI for anxiety and the BDI for depression. USA was entered as the referenced category for both meta-regressions for the country variable. We did not conduct a meta-analysis on intervention effects due to there being too few studies (less than 10; Watson et al., 2016).

Interviews were conducted with four research experts in self-compassion (see Table 2)—Neff, Bluth, Ferrari and Gilbert. A Youth Advisory Group (YAG) of 20 young people aged 14–24 (see Table 3 for details) were consulted at two stages (see Table 4). The first consultation with youth was conducted in July 2020. In the second consultation (August 2020), feedback was sought on a summary of research findings (see Supplementary Material Table 1) which was emailed 5 days prior to the interview. Young people were given an AUD$95 Amazon voucher. The Youth Advisory Group was recruited in Australia via social media, word of mouth and email, and did not report having previously engaged in self-compassion interventions. Consent was received from people over 16 years, or from their parents if they were under 16. Interviews were conducted on Zoom either individually or in focus groups, depending on preference. The interviews were conducted by two research assistants, one of whom was an undergraduate psychology student, and the other a registered clinical psychologist with extensive experience in engagement of young people in clinical practice. The interviews were structured and included all questions in Table 4, which were asked in both the individual interviews and focus groups.

**Table 2:** Interview questions with research experts

Interview questions	
1	How do you define self-compassion?
2	In your experience, how is self-compassion experienced by young people?
3	In your experience, how is self-compassion relevant for young people with or at-risk of depression and anxiety?
4	Do you consider self-compassion to be an active ingredient in interventions for prevention or treatment of anxiety and depression in young people? Why or why not?
5	Do you think self-compassion interventions should be modified for young people aged 14–24 compared to treatments for adults? If so, how?
6	What would you say are the most important components of self-compassion treatments relevant to young people?
7	What do you think are the best formats for self-compassion interventions in young people?
8	Do you have any further opinions you could offer to help in this research?

Interviews lasted 20–35 min. Experts were given a AUD$25 Amazon voucher

**Table 3:** Youth advisory group demographics, interview type and length

YAG demographics			
Gender	Number	Percentage	
Male	6	30%	
Female	12	60%	
Non-binary	2	10%	
Age	Range	Mean	SD
	14–24	M = 18.85	SD = 3.63
Lived experience anxiety/depression	Confirmed lived experience	Drawing on friends’ experience	Unknown
	N = 11	N = 6	N = 3

Interview type	Focus group	Individual
Phase 1	N = 9	N = 11
Phase 2	N = 15	N = 5
Interview length	45–90 min (M = 57.04 min)	15–50 min (M = 24.13 min)

*YAG* Youth Advisory Group, *SD* Standard Deviation

**Table 4:** Interview questions with youth advisory group

Phase 1	Self-compassion in relation to lived experience of anxiety and depression
1	Is self-compassion something you have heard of before? If so, in what context?
2	What do you think self-compassion is?
3	How do you think self-compassion relates to your own, or your friends, experiences of symptoms of depression (feeling down) or anxiety (feeling worried all the time)?
4	Do you think there are any difficulties with the idea of self-compassion? If so, please tell me more? (For example, do you think there are any groups of people who might struggle with the idea of self-compassion?)
5	Do you think a programme aimed at increasing self-compassion might help to prevent anxiety and depression? If so, how?
6	How do you think self-compassion can be useful in psychological interventions? Why?
7	In what format would you prefer to receive an intervention programme? (For example, online guided or unguided, face to face, self-help booklet etc.?)
8	Do you think an intervention aimed at increasing self-compassion (being kind to yourself) would be helpful to you or other young people with anxiety and depression?
9	What do you think researchers should consider when thinking about self-compassion, anxiety and depression in young people?

Phase 2	Feedback on summary of research findings
1	How relevant and useful did you think the summary of the research was to your own or your friends’ experiences of symptoms of anxiety and depression?
2	How do you think self-compassion could be used as a treatment for symptoms of anxiety and depression in young people?
3	Does an intervention aimed at increasing self-compassion appeal to you?
4	Do you have any further comments we should consider in what appeals to you or would be useful regarding self-compassion as a treatment for anxiety and depression in young people?

Interviews with the researchers and youth were transcribed and the results were analysed following guidelines for thematic analysis of Braun and Clarke (2006). The six steps of thematic analysis outlined by Braun and Clarke (2006) were followed. For example, the transcripts were examined by the author who conducted the qualitative analysis (JD) and the main ideas were described that emerged with key words, then ideas were grouped into overarching themes. To ensure quality and rigour of the process, initial themes were discussed with two senior authors (SE and CR) before being finalised. The researcher who conducted the thematic analysis (JD) selected quotations from transcripts that best represented the theme in question as per Braun and Clarke’s (2006) recommendations.

## Results

### Search Summary and Study Characteristics

**Fig. 1:**
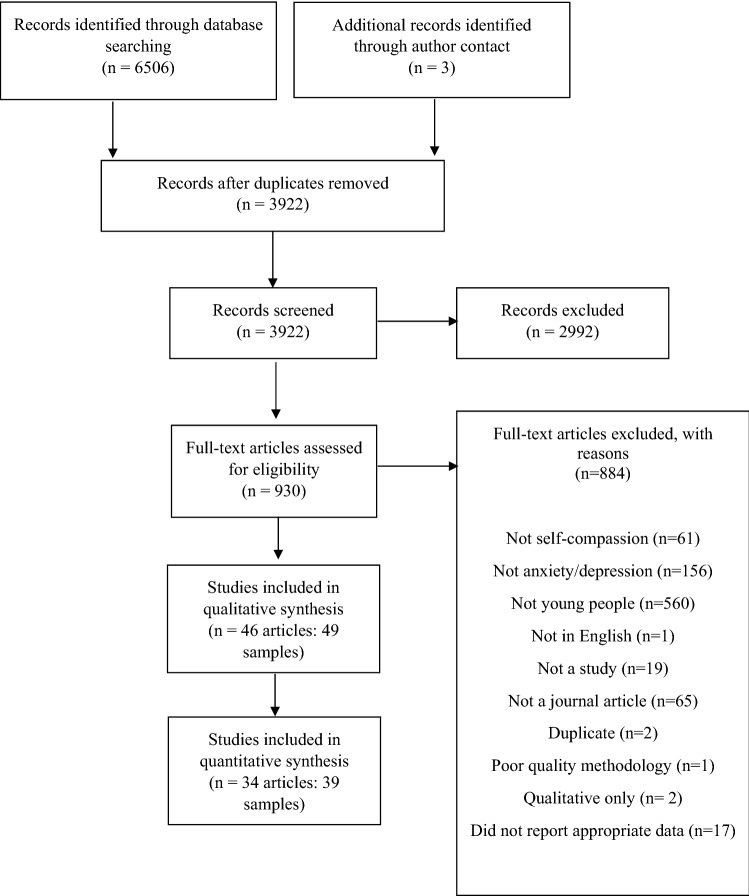
PRISMA flowchart outlining the search process

The search yielded 6506 results. After the removal of duplicates, 3922 studies were screened by title and abstract. Full-text screening was undertaken for 930 studies, as outlined in Fig. 1. A total of 49 studies (46 articles) met inclusion criteria. Just under half of the studies were from the USA (n = 23), thereafter a range of countries including China (n = 5), Japan (n = 5) and (n = 2) in Australia, Belgium, Canada, Portugal. There were single studies in 8 other countries (see Table 5). Pooled sample size was 21,792 (~ 60% female).

**Table 5:** Study characteristics of all studies included in the systematic review

First author	Q^a^	Sample size	Sample	Mean age(range/SD)	%female	Country	Study type	Measure Anxiety	Measure Depression
Arch et al. (2014)	1	105	University students	19.53 (SD = 1.88)	100%	USA	RCT	STAI, SPS, SIAS	
Arimitsu (2016)	1	40	University students with low self-compassion	23.25 (I) (SD = 7.41) 19.42 (C) (SD = 1.08)	75%	Japan	RCT	STAI	BDI-II
Bluth et al. (2015)	1	28	Middle school and high school students	NR (10–18)	57%	USA	NRS	PSS	PSS
Bluth et al. (2016)	1	34	High school students	NR (14–17)	74%	USA	RCT	STAI	SMFQ
Burke et al. (2020)	1^b^	72	University students with mild-moderate depressive symptoms	19.33 (SD = 1.20;18–23)	65%	USA	Prospective	STAI	BDI
Ko et al. (2018)	1^b^	41	University students	19.78 (SD = 1.36; 18–22	66%	USA	RCT	STAI	CES-D
Polizzi et al. (2019)	1	131	University students	18.96 (SD = 1.00)	57%	USA	RCT	STAI	CES-D
Smeets et al. (2014)	1	52	University students	19.96 (SD = 1.33)	100%	Netherlands	RCT	Penn State Worry	
Arimitsu and Hofmann (2015)	2	S1:231S2: 233	University students	19.11(SD = 0.88; S1), 19 (SD = 0.97; S2)	S1: 43% S2: 28%	Japan	Cross-sectional	STAI	BDI-II
Asano et al. (2020)	2	279	University students	19.35 (SD = 3.26; 18–23)	69% (6 NR)	Japan	Cross-sectional	DASS-15	DASS-15
Bluth et al. (2017)	2	765	High school students	NR (11–19)	53%	USA	Cross-sectional	STAI	SMFQ
Castilho et al. (2017)	2	1101	Community adolescents	15.94 (SD = 1.21; 14–18)	57%	Portugal	Cross-sectional		CDI
Cunha et al. (2016)	2	3165	High school students	15.49 ( SD = 1.59; 12–19)	54%	Portugal	Cross-sectional	DASS-21	DASS-21
Ferrari et al. (2018)	2	541	High school students	14.1 (SD = NR; 12–15)	82%	Australia	Cross-sectional		SMFQ
Ferrari et al. (unpublished)	2	951	High school students	13.69 (SD = 0.73; 12–16)	46%	Australia	Cross-sectional	SCAS	
Galla (2016)	2	132	Community adolescents	16.76 (SD = 1.48; 13.8–20.3)	61%	USA	Longitudinal		CES-DC
Garcia-Campayo et al. (2014)	2	268	University students	20.54 (SD = 2.11)	60%	Spain	Cross-sectional	STAI	BDI-II
Ghorbani et al. (2012)	2	238	Iranian Muslim University students	21.60 (SD = 2.40)	52%	Iran	Cross-sectional	Costello and Comrey Anxiety scale	Costello and Comrey Depression Scale
Gill et al. (2018)	2	316	High school students	14.77 (SD = 0.89; 14–18)	46%	UK	Cross-sectional	SCARED, SPIN	
Hall et al. (2013)	2	182	University students	NR	77%	USA	Cross-sectional		BDI-II
Hou et al. (2020)	2	578	University students	20.30 (SD = 1.29; 17–24)	48%	China	Cross-sectional		BDI-I
Kemper et al. (2016)	2	29	Hospital migraine outpatients	14.80 (SD = 2.00; 12–18)	69%	USA	Cross-sectional	PROMIS Short-Form Paediatric Anxiety Scale	PROMIS Short-Form Paediatric Depression Scale
Lahtinen et al. (2019)	2	2383	High school students	NR(16–18)	52%	Finland	Cross-sectional		BDI-II-R
Lathren et al. (2019)	2	1057	High school students	14.70 (SD = NR)	65%	USA	Cross-sectional	STAI	SMFQ
Luo et al. (2019)	2	1453	University students	19.58 ( SD = 1.09; 17–23)	99%	China	Cross-sectional		GADS
Mingkwan et al. (2018)	2	390	University students	NR(18–22)	63%	Thailand	Cross-sectional	TMHQ	THMQ
Miron et al (2016)	2	377	University students	19.12 (SD = 1.73)	64%	USA	Cross-sectional		DASS-21
Neff (2003b)	2	S1:391S2:232	University students	20.91 (SD = 2.27;S1) 21.31 (SD = 3.17; S2)	S1: 42% S2: 62%	USA	Cross-sectional	STAI	BDI-II, Zung self-rating depression scale
Neff et al (2008)	2	568	University students American, Thai, Thaiwanese	24.1/19.8/20.5 (SD = NR)	59%	USA	Cross-sectional		Zung Self-Rating Depression Scale
Podina et al. (2015)	2	187	University students	23.62 (SD = 5.64)	81%	USA	Cross-sectional		BDI-II
Rabon et al. (2018)	2	356	Rural university students	21.44 (SD = 5.16)	68%	USA	Cross-sectional		CES-D
Raes (2010)	2	271	University students	18.14 (SD = 1.25)	79%	Belgium	Cross-sectional	STAI-T	BDI-II
Stefan et al. (2018)	2	63	University students	18.76 (SD = 0.43)	75%	Romania	RCT	LSAS-SR	
Stephenson et al. (2018)	2	184	University students	19.20 (SD = 1.50)	52%	USA	Cross-sectional	Costello and Comrey Depression and anxiety scales	Costello and Comrey Depression and anxiety scales
Stutts et al. (2018)	2	462	University students	NR (18–20)	72%	USA	Longitudinal	SCL-90	SCL-90
Svendsen et al. (2017)	2	277	University students	22.9 (SD = 3.6)	56%	Norway	Cross-sectional		SCL-90-R
Tanaka et al. (2011)	2	117	Adolescents involved with child welfare	18.10 (SD = 1.00; 16–20)	61%	Canada	Cross-sectional		CES-D
Terry et al. (2013)	2	119	University students	NR	47% (5 NR)	USA	Longitudinal		CES-D
Van der Gucht et al. (2018)^c^	2	408	High school students	15.40 (SD = 1.20)	58%	Belgium	RCT	DASS-21	DASS-21
Wilson et al. (2020)	2	228	University Students	19.84(SD = 2.75)	76%	USA	Cross-sectional		CES-D
Woodruff et al. (2014)	2	147	University students	NR (17–23)	71%	USA	Cross-sectional	BAI	BDI
Yamaguchi et al. (2014)	2	S1:1200 S2: 420	University students: Japanese and American	S1 = 19.6 (SD = 0.97); S2 = 21 (SD = 3.62)	S1: 36% S2: 76%	S1: Japan S2: USA	Cross-sectional		CES-D
Zeifman et al. (2019)	2	130	University students	21.04 (SD = 6.30; 17–60)	83%	Canada	Cross-sectional		DASS-21
Zhang and Wang (2019)	2	112	University students with physical disabilities	21.24 (SD = 2.66)	42%	China	Cross-sectional		CES-D
Zhou et al. (2013)	2	418	University students	19.17 (SD = 1.04; 16–23)	74%	China	Cross-sectional		HDSQ
Zhou et al. (2017)	2	330	University students	19.90 (SD = 1.30; 16–24)	37%	China	Cross-sectional	BAI	BDI

*N* sample size, *NR* not reported, *NA* not applicable, *RCT* Randomised Control Trial, *NRS* Non-Randomised Control Study, *BAI* Beck Anxiety Inventory, *BDI-II* Beck Depression Inventory-II, *CDC* Centre of Disease Control, *CDEQ* Children’s Depressive Experiences Questionnaire, *CDI* Children’s Depression Inventory, *CESD* Centre for Epidemiological Studies Depression Scale, *DASS-15* Japanese version of DASS-21, *DASS-21* Depression Anxiety and Stress Scale, *GAD-II* Generalized Anxiety Disorder Screener, *GADS* Goldberg Anxiety and Depression Scale, *LSAS-SR* Liebowitz Social Anxiety Scale, *HDSQ* Hopelessness Depression Symptom Questionnaire, *PROMIS* Patient-Reported Outcome Measurement Information System, *PSS* The Perceived Stress Scale, *SCAS* Spence Children’s Anxiety Scale, *SCARED* Screen for Child Anxiety Related Emotional Disorders, *SCL-90* Symptom checklist, *SIAS* Social interaction Anxiety Scale, *SMFQ* The Short Mood and Feeling Questionnaire, *SPIN* Social Phobia Inventory, *SPS* Social Phobia Scale, *STAI* Spielberger State-Trait Anxiety Inventory, *TMHQ* Thai mental health questionnaire

^a^Related review question

^b^Also included in review question 2

^c^Inconsistency in reporting of sample size

### Quality Ratings

Quality ratings ranged from 28.57 to 90.91%, using the National Institutes of Health (2014) Quality Assessment tools, see Table 6. For the purposes of the meta-analysis for question 2, studies were coded based on quality as follows: (50% > poor, 51–74.99%- fair, and 75% < good).

**Table 6:** Quality assessment ratings of studies in the systematic review using the NIH Quality Rating Tool for Cross-Sectional and Observational Studies

Study Authors	1	2	3	4	5	6	7	8	9	10	11	12	13	14	Rating (%)
Arch et al. (2014)^a^	•	~	~	•		•	•	•	•	~	•		•	•	64.29
Arimitsu and Hofmann (2015)	•		~	•		~	•	~	•		•	~	~		55.56
Arimitsu (2016)^a^	•	~	~		~	~	•	•	~	~	•	•	•	•	50.00
Asano et al. (2020)	•	•	~	•		~	•	~	•		•	~	~		66.67
Bluth et al. (2015)^a^						~	•	~	•	~	•	•	•	•	42.86
Bluth et al. (2016)^a^		~	~	•	~	•	•	•	•	~	•	•	•		57.14
Bluth et al. (2017)	•	•		•	•	~	•	•	•		•	~	~	•	90.00
Burke et al. (2020)^a^				~	~	~	•	~	•	~	•		•	•	35.71
Castilho et al. (2017)	•		~	•		~	•	~	•	•	•	~	~		66.67
Cunha et al. (2016)	•	•	~	•		~	•	~	•		•	~	~		66.67
Ferrari et al. (2018)	•	•	~		•	~	•	~	•		•	~	~	•	77.78
Ferrari et al. (unpublished)	•	•			•	~	•	~	•	•	•	~	~	•	80.00
Galla (2016)	•	•	•	•		~	•	~	•	•	•	~	•	•	90.91
Garcia-Campayo et al. (2014)	•	•	~	•	•	~	•	~	•		•	~	~		77.78
Ghorbani et al. (2012)	•	•	~	•		~	•	~	•		•	~	~	•	77.78
Gill et al. (2018)	•	•	~	•	•	~	•	~	•		•	~	~	•	88.89
Hall et al. (2013)	•		•	•	•	~	•	~	•		•	~	~		70.00
Hou et al. (2020)	•	•	~	•		~	•	~	•		•	~	~	•	77.78
Kemper et al. (2016)	•	•	~	•		•	•		•	•	•	~	~		72.73
Ko et al. (2018)^a^	•	•	•	~	~	•	•	•	•	~	•	~	•	•	71.43
Lahtinen et al. (2019)	•	•	~	•		~	•	~	•	•	•	~	~	•	88.89
Lathren et al. (2019)	•	•	•	•		~	•	~	•		•	~	~	•	80.00
Lihua et al. (2017)	•	•	~	•		~	•	~	•		•	~	~		66.67
Luo et al. (2019)	•	•		•		~	•	~	•		•	~	~		60.00
Mingkwan et al. (2018)	•	•	~	•	•	~	•	~	•		•	~	~		77.78
Miron et al. (2016)	•	•	~	•		~	•	~	•	•	•	~	~		77.78
Neff (2003b)	•	•	~			~	•	~	•		•	~	~	•	66.67
Neff et al. (2008)	•	•	~			~	•	~	•		•	~	~	•	66.67
Podina et al. (2015)	•		~			~	•	~	•		•	~	~	•	55.56
Polizzi et al. (2019)^a^		~	~			~	~	~	•	~	•	•	•	•	35.71
Rabon et al. (2018)	•		~			~	•	~	•		•	~	~		44.44
Raes (2010)	•	•	~	•		~	•	~	•		•	~	~	•	77.78
Smeets et al. (2014)^a^	•	•	~	~	~	•	•	•	~	~	•	~	•	•	57.14
Stephenson et al. (2018)	•		~	•		~	•	~	•		•	~	~	•	66.67
Stefan (2019)	•	•			•	~	•	~	•	•	•	~	~	•	80.00
Stutts et al. (2018)	•		~			~	•	~	•	•	•	~	~	•	66.67
Svendsen et al. (2017)	•	•	~	•		~	•	~	•		•	~	~		66.67
Tanaka et al. (2011)	•	•	•	•		~	•	~	•		•	~	~	•	80.00
Terry et al. (2013)	•	•	~			~	•	~	•	•	•	~	~	•	77.78
van der Gucht et al. (2018)^a^		•	~	~	~	~	~	~	~	~	•	~	•	•	28.57
Wilson et al. (2020)	•	•	~	•		~	•	~	•		•	~	~	•	77.78
Woodruff et al. (2014)	•		~	•		~	•	~	•		•	~	~		55.56
Yamaguchi et al. (2014)	•	•	~	•	•	~	•	~	•		•	~	~		77.78
Zeifman et al. (2019)	•		~	•		~	•	~	•		•	~	~	•	66.67
Zhang and Wang (2019)	•	•	~	•		~	•	~	•		•	~	~	•	77.78
Zhou et al. (2013)	•		•			~	•	~	•		•	~	~		50.00

Study quality was assessed using the NIH Quality Assessment tools; 14-item scales to evaluate internal validity (National Institutes of Health, 2014). Scores were calculated as the average of applicable items and are represented as a percentage. Appropriate reporting for items in each article is indicated with a dot (i.e., bullet points refer to the criteria being met)

^a^Studies were assessed using the NIH Quality Rating for Controlled Interventions Scale; ~ Item was deemed Not Applicable (NA), Cannot Determine (CD), or Not Reported (NR)

Research Questions:

1. Do self-compassion interventions demonstrate efficacy for symptoms anxiety and depression in young people?
  As seen in Table 5, eight studies (six RCTs, two open trials) examined self-compassion interventions in young people. Two studies were with adolescents (Bluth et al., 2015, 2016), and six with university students. Six of the eight studies (Armitsu, 2016; Bluth et al., 2015, 2016; Burke et al., 2020; Ko et al., 2018; Smeets et al., 2014) were group-based interventions, the remaining two interventions were individual lab-based studies where participants listened to the intervention on a computer (Arch et al., 2014; Polizzi et al., 2019). In terms of who provided the interventions, five of the eight studies were delivered by therapists who were trained in self-compassion (Armitsu, 2016; Bluth et al., 2015, 2016; Burke et al., 2020; Smeets et al., 2014), two were delivered by researchers in lab-based studies (Arch et al., 2014; Polizzi et al., 2019), and one study was delivered by a university lecturer (Ko et al., 2018). Of the eight studies, only two were targeted at either low-self-compassion (Arimutsu, 2016) or mild-moderate depressive symptoms (Burke et al., 2020), the remainder were not specifically reported as prevention or treatment studies. Of the eight intervention studies, three had relatively large sample sizes suggesting they were adequately powered (Arch et al., 2014; Burke et al., 2020; Polizzi et al., 2019), given the relatively small sample sizes of the remainder (see Table 5), it is likely that they were underpowered. See supplementary Tables 4, 5, 6, 7 for detailed examples of intervention content for five of the eight included studies (Arch et al., 2014; Arimitsu, 2016; Bluth et al., 2015, 2016; Burke et al., 2020), not all interventions are detailed due to consideration of length.
  Bluth et al. (2015) examined a six-session self-compassion group intervention in community adolescents not selected for elevated anxiety/depression. This was an open trial but found significant reductions in stress and increases in self-compassion. A further waitlist-controlled crossover trial in adolescents conducted by Bluth et al. (2016) found the intervention group demonstrated medium effect size reductions in anxiety and depression.
  Some studies examining university students included very brief interventions. Arch et al. (2014) examined the impact of a brief, two-session self-compassion intervention consisting of meditation practices in female university students, and found significantly decreased anxiety, as well as reduced sympathetic and cardiac parasympathetic responses to a social stress test, compared to attention placebo and control conditions. In contrast, Polizzi et al. (2019) found no impact of a two-session loving-kindness meditation intervention in university students on worry or depression and concluded the intervention was too brief (12 min per session). Smeets et al. (2014) examined a three-session self-compassion group intervention with female university students, and found compared to an active control, the intervention group demonstrated a medium effect size reduction in rumination, however there was no significant change in worry or negative affect. However, in an open trial evaluating a four-session group self-compassion intervention with university students with elevated depressive symptoms in the UK, Burke et al. (2020) found pre-post reductions in anxiety and depression.
  Two RCTs with university students examined longer interventions. Arimitsu (2016) examined a seven-session self-compassion intervention (including loving-kindness meditation, mindfulness training and compassionate mind training) with Japanese university students who had low self-compassion on the total score of the SCS (Neff, 2003a). Significant decreases were observed in depressive and anxious symptoms compared to controls, maintained at three-month follow-up. However, with 28 participants, this study was underpowered. Ko et al. (2018) found an intervention in university students consisting of 30 seminars on self-compassion had no impact on anxiety or depression.
2. Is self-compassion associated with anxiety and depression in young people?
  Forty articles reported on the associations between self-compassion and anxiety and/or depression (see Table 5). Articles were mostly cross-sectional (n = 32) with a small number of longitudinal studies (n = 5) and three controlled trials reporting relevant correlations at baseline. The majority of studies were with university students (n = 28). Seven studies comprised high school student samples and three were community samples. The remaining two samples were hospital outpatients (treated for migraine), and adolescents involved with child protection/welfare. Thirty-four articles with 39 samples provided sufficient data to calculate effect sizes for the associations between self-compassion (total score) and anxiety and/or self-compassion (total score) and depression. Twenty-one effect sizes were pooled for the anxiety association and 37 for depression association.
  Across studies, significant negative correlations between self-compassion and anxiety were reported, ranging from *r* = − 0.19 to − 0.75, with a moderate-to-large, pooled effect size [Pooled *r* = − 0.49, 95% CI (− 0.57, − 0.42), p < 0.001]. However, heterogeneity was high [Q (20) = 273.37, p < 0.001, I^2^ = 93.42%], thus, we ran a meta-regression entering study quality, country, and measure of anxiety as moderators (Table 7). These three variables accounted for the heterogeneity across studies [Q (5) = 7.60, *p* = 0.18, I^2^ = 0.00%]. Specifically, high study quality accounted for a significant amount of variance in effect size and was associated with significantly smaller effect sizes than low study quality. Second, the DASS, BAI, and the SCL accounted for significant variance in effect sizes and were associated with significantly larger effects than the STAI. Lastly, studies taking place in Japan, Portugal, and Belgium accounted for a significant amount of variance in effect sizes and were associated with significantly larger effect sizes compared to studies taking place in the USA. However, these moderation effects should be considered with caution, due to the low number of studies for each category.

**Table 7:** Meta-regressions for the associations between self-compassion and anxiety (left), and self-compassion and depression (right)

Anxiety				Depression			
	B	SE	95% CI [lower, Upper]		B	SE	95% CI [lower, Upper]
Intercept	− 0.69***	0.065	[− 0.82, − 0.57]	Intercept	− 0.52***	0.043	[− 0.07, 0.10]
Medium Quality^a^	0.10	0.068	[− 0.03, 0.24]	Medium Quality^a^	− 0.03	0.038	[− 0.01, 0.14]
High Quality^a^	0.17*	0.065	[0.04, 0.29]	High Quality^a^	0.02	0.034	[− 0.15, − 0.01]
DASS^b^	0.52***	0.058	[0.40, 0.63]	DASS^d^	0.07	0.054	[− 0.16, 0.05]
SCAS^b^	0.06	0.041	[− 0.02, 0.14]	SMFQ^d^	− 0.08*	0.037	[− 0.07, 0.08]
Costello^b^	0.11	0.068	[0.02, 0.24]	CDI^d^	− 0.05	0.070	[− 0.12, 0.15]
SCARED^b^	− 0.02	0.056	[− 0.13,0.09]	CES − D^d^	0.01	0.069	[− 0.13, 0.14]
PROMIS^b^	0.18	0.177	[− 0.17, 0.53]	ZUNG^d^	0.02	0.052	[0.06, 0.27]
BAI^b^	0.19**	0.079	[0.04, 0.35]	Costello^d^	0.01	0.146	[− 0.40,0.17]
SCL^b^	0.32***	0.049	[0.23, 0.42]	SCL^d^	0.17**	0.031	[0.06, 0.27]
China^c^	0.17	0.093	[− 0.01, 0.35]	PROMIS^d^	− 0.11	0.043	[− 0.40, 0.17]
Japan^c^	− 0.13***	0.037	[− 0.20, − 0.05]	Japan^c^	0.13***	0.061	[0.07, 0.19]
Portugal^c^	− 0.31***	0.063	[− 0.43, − 0.18]	Portugal^c^	− 0.01	0.068	[− 0.10, 0.07]
Spain^c^	− 0.01	0.060	[− 0.13, 0.10]	Australia^c^	− 0.18**	0.095	[− 0.30, − 0.06]
Belgium^c^	− 0.23***	0.047	[− 0.32, − 0.14]	Spain^c^	0.08	0.038	[− 0.06, 0.21]
Iran^c^	0.18	0.097	[− 0.02, 0.37]	Iran^c^	0.14	0.036	[− 0.05, 0.33]
				China^c^	0.07	0.089	[− 0.00, 0.14]
				Finland^c^	0.24	0.092	[0.17, 0.31]
				Thailand^c^	0.01	0.052	[− 0.16, 0.18]
				Taiwan^c^	− 0.07	0.070	[− 0.25, 0.11]
				Belgium^c^	− 0.04	0.066	[− 0.14, 0.06]
				Norway^c^	− 0.20**	0.043	[− 0.34, − 0.06]
				Canada^c^	0.13	0.038	[0.00, 0.26]

*BAI* Beck Anxiety Inventory, *BDI* Beck Depression Inventory, *CDI* Children's Depression Inventory, *CES-D* Centre for Epidemiological Studies Depression Scale, *Costello* Costello and Comrey Anxiety and Depression scales, *DASS* Depression Anxiety and Stress Scale, *PROMIS* Patient-Reported Outcome Measurement Information System, *PSS* The Perceived Stress Scale, *SCAS* Spence Children’s Anxiety Scale, *SCARED* Screen for Child Anxiety Related Emotional Disorders, *SCL* Symptom checklist, *SIAS* Social interaction Anxiety Scale, *SMFQ* The Short Mood and Feeling Questionnaire, *STAI* Spielberger State-Trait Anxiety Inventory, *ZUNG* Zung self-rating depression scale

*p < 0.05

**p < 0.01

***p < 0.001

^a^Low quality (reference group)

^b^STAI (reference group)

^c^USA (reference group)

^d^BDI (reference group)
  The results for depression were similar, with significant negative correlations between self-compassion and depression, (*r* = − 0.30 to − 0.63), with a moderate-large, pooled effect size [Pooled *r* = − 0.50, 95% CI (− 0.53, − 0.47), *p* < 0.001]. However, again, heterogeneity was high [Q (36) = 255.16, *p* < 0.001, I^2^ = 78.13%]. Country and measure of depression accounted for majority of the heterogeneity across studies [Q (14) = 23.99, *p* = 0.046, I^2^ = 10.32%: Table 7]. Specifically, the SMFQ and SCL accounted for a significant amount of variance in effect sizes. The SMFQ was associated with significantly larger effect sizes, whereas the SCL was associated with significantly smaller effect sizes, compared to the BDI. Second, studies taking place in Australia, Norway, and Japan accounted for significant variance in effect sizes. Studies taking place in Australia and Norway were associated with significantly larger effect sizes, whereas studies taking place in Japan were associated with significantly smaller effect sizes, compared to studies taking place in the USA. However, these moderation effects should be considered with caution, due to the low number of studies for each category.
3. How do young people experience self-compassion and how does this relate to their experience of anxiety and depression?

Feedback from Youth Advisory Group (stage 1):

Numerous themes emerged from the first advisory group (see Table 8 and Supplementary Table 2).

**Table 8:** Themes and sub-themes from a thematic analysis of self-compassion and how it relates to experiences of anxiety and depression (stage 1 consultations)

Themes	Sub-themes
Self-compassion as the opposite of self-criticism	SC to reduce self-criticism and disengage from negative thoughts
	SC means having to sacrifice your goals and achievements
	Stigma that SC means you are weak/lazy
	Hard to get into the SC mindset, particularly in stressful/high pressure moments where self-criticism is high
	Self-criticism means young people treat themselves less compassionately than they do others
Young people’s understanding and awareness of self-compassion	Limited exposure to the concept of SC and need to raise awareness
	Assumption it is the same as self-care
	Increased SC can help reduce anxiety and depression
	SC dependent on family background/relationships/culture
Preferences for SC programmes	SC programs are needed and would be helpful
	SC programs tailored to person’s preferred format and to specific groups (eg. Culture, LGBTIQ +)
	Researchers should be aware of how certain words/behaviours come across to young people

*SC* self-compassion, *LGBTIQ* + Lesbian, Gay, Bisexual, Trans, Intersex and Queer plus

#### Theme 1. Self-compassion as the opposite of self-criticism

Nearly all the young people (95%) identified that self-criticism acted as a barrier to practicing self-compassion due to fears it would lead to decreased performance and *mean having to sacrifice on their goals and achievements.* One young person said self-compassion would “*prevent her from trying to strive for excellence” (Female, 24 years old, Participant 8).* Another said: *“It feels counterintuitive to try to have compassion for yourself when there’s another part of you that’s like if you just bully yourself enough maybe you’ll get up and get on with the day”. (Non-binary, 19 years old, Participant 18).*

Some young people (35%) associated *self-compassion with being “weak” or “lazy”,* others said other concepts like self-care and self-love had a “cringe” factor, for example: *“I think definitely as a teenager it’s a little bit stigmatised as you’re weak or it’s a little bit cringy maybe to try and do these things for yourself”. (Female, 16 years old, Participant 12).*

Young people said it was *difficult to practice self-compassion in moments of high distress*. Although they recognised its benefits, they identified that a significant mindset shift would be required to switch from an experience of anxiety and depression, characterised in large part by self-criticism, to being self-compassionate.

Young people talked about self-compassion as a method of coping with failure and *reducing self-criticism*, defining self-compassion as “*being compassionate towards your own failure”.* Young people also identified self-compassion as a *helpful tool to disengage from negative thoughts* and improve self-worth. Some said having more self-compassion would mean being “*less likely to be anxious and depressed*” and would “*make it easier to control anxiety*”. Many young people (75%) identified that self-compassion would reduce negative self-talk, for example: *“If you want to judge yourself fairly then you have to be kind to yourself because you can't judge yourself fairly if you're always being harsh on yourself.” (Male, 17-year-old, Participant 6).*

#### Theme 2. Young people’s understanding and awareness of self-compassion

The majority (85%) of young people had *limited exposure to self-compassion*, five *assumed self-compassion to be synonymous with or similar to self-care*. Young people stressed the importance of *raising awareness of self-compassion*:

“I think you need to get the concept out there, none of us really knew about what self-compassion was…” (Female, 16 years old, Participant 12).

From their understanding of self-compassion, young people largely felt it would *help reduce and prevent symptoms of anxiety and depression*.

“If you learn how to have self-compassion, and learn how to treat yourself better, it can probably help with symptoms of anxiety and depression.” (Male, 14 years old, Participant 2).

Young people identified that *family background, culture and relationships* played a large part in awareness of self-compassion, and ability to practice it:

“Asian people, we grow in families that often are more critical and so with a family background like that, understandably you develop a habit of criticising yourself, and it's harder for us to practice self-compassion.” (Female, 24 years old, Participant 8).

#### Theme 3. Preferences for self-compassion programmes

Most young people (90%) felt *self-compassion programmes would be helpful* to prevent and treat anxiety and depression, especially in decreasing self-critical thoughts. One 22-year-old male said a programme would help *“as a framework to allow someone to reframe their thought process” (Participant 9)*. Preferences for intervention formats varied. Some chose an online programme or an app, while others preferred face-to-face interventions—either one-on-one or in a group. A common theme was that young people wanted to feel they could apply strategies to suit their own individual challenges. Rather than a “cookie-cutter” solution, they wanted to feel that the programme was targeted, for example:

"I think [researchers] should consider that, it would be quite different for different people, like they might struggle with different things depending on their experiences." (Female, 14 years old, Participant 5).

Young people suggested *tailoring programmes to specific minority groups*, including cultural minorities and LGBTQIA + individuals. Young people also warned *researchers should be aware of how certain words and behaviours come across*. The word “intervention”, for example, was associated with drug or alcohol interventions in movies and television. Participants also warned against programmes where adults try to sound “cool”, as the attempt at relatability usually falls short.

“…terms like self-compassion, self-love, mindfulness, they sort of, they feel like buzzwords to people, and in a way have negative connotations because of that.” (Female, 21 years old**,** Participant 13).

(4) How do experts in self-compassion research view self-compassion as an active ingredient in youth?

Research experts said self-compassion is the same regardless of age, however developmental differences might mean the concept needs to be explained differently depending on age. They believed self-compassion could help young people experiencing anxiety by providing a feeling of safety and preventing overidentification and rumination. In relation to depression, self-compassion could help improve self-acceptance.

“When you start being kind to yourself and realising that you’re imperfect…that everyone’s imperfect, that life is imperfect, and you stop being attached to things being a certain way and you accept yourself as you are, then you stop judging yourself so much, you start accepting yourself more, and that directly reduces depression.” Neff (2020).

Self-compassion was believed to work as an active ingredient by targeting mechanisms like self-criticism, central to anxiety and depression.

“…rumination and self-criticism are core maintaining factors for (depression)… I think self-compassion directly targets the mechanisms that maintain depression.” Ferrari (2020).

A key barrier was the misinterpretation of self-compassion as an act of laziness, or that self-criticism is necessary to push oneself, for example:

“[Young people] are raised with this idea that in order to be successful… they have to be hard on themselves, they have to beat themselves up. So the idea that they can actually be kind to themselves and achieve what they’re supposed to achieve in life—meaning getting into a good college, eventually getting a good job—it’s eye opening to them that they can do that”. Bluth (2020).

Gilbert raised the importance of distinguishing between self-compassion and self-kindness. Unlike self-kindness, which focuses primarily on the promotion of wellbeing, self-compassion requires *courage* and *wisdom*—courage to recognise and engage with your own pain and suffering rather than turning away from it, and wisdom to differentiate between helpful and harmful behaviours in moments of distress.

“We find that clinically, self-compassion enables people to develop the courage and strengths they need, along with the capacity to think empathically and gain insight into ‘what my pain is about’, learning to tolerate those feelings and make sense of them, and then working out wise ways of dealing with them. While obviously useful and important, kindness does not involve that courageous and wise engagement with suffering. So when you really dig into the issue of suffering, we have to descend and enter into it not away from it, that’s compassion”. Gilbert (2020).

Experts said group interventions work particularly well for young people, and highlighted the value of psychoeducation for young people in their late teens and early 20s.

(5) How do young people view the review, and what are their recommendations for interventions focused on self-compassion?

Three themes emerged in the second consultations (see Table 9 and supplementary Table 3).

**Table 9:** Themes and sub-themes from a thematic analysis of young people’s response to a summary of research findings (stage 2 consultations)

Themes	Sub-themes
Research and expert opinions are relevant to lived experience of anxiety and depression	Focusing on reducing self-criticism as opposed to increasing self-kindness resonates with young people
	Framing self-compassion in terms of courage and wisdom makes it more appealing
	Having research to back mental health programmes helps them stand out from the sea of “self-help” methods advertised online
The novelty of SC means it might take time for it to garner real credibility among young people	Clarity is needed around the definition of key terms and the practical aspects of self-compassion programmes
	Young people need to become more familiar with SC to be able to trust it and engage with it
	Knowing more about self-compassion increases likelihood of participating in a programme
SC programs are appealing but initial engagement can be difficult	While information is relevant, it is unlikely to be consumed unless there has been previous engagement
	Information needs to be dynamic, brief, tailored to individuals, and promoted on the right platforms

*SC* self-compassion

#### Theme 1. Research and expert opinions were relevant to lived experience of anxiety and depression

Research findings about *changes in depression and anxiety being related to changes in self-criticism, rather than self-kindness,* were particularly relevant for young people. Six young people (30%) were interested in this finding specifically. They said the idea that reduced self-criticism is related to less anxiety and depression made sense. A point raised by Gilbert, which *described self-compassion in terms of courage and wisdom,* also strongly resonated with young people. Young people found this idea noteworthy and engaging, and felt framing a programme in this way—rather than with a focus on “self-kindness” and “self-compassion”—would be more appealing.

“I think that for me if someone would say, you should practice self-compassion, I would be like, ah, I don't know. But if someone would relate it to wisdom and you’d be a lot wiser and more courageous, I think that would appeal to me more.” (Female, 21 years old, Participant 10).

#### Theme 2. The relative novelty of self-compassion means it might take time for it to garner credibility among young people

Some of the young people (55%) stressed the importance of differentiating between terms like self-compassion, self-care, self-love, self-kindness and mindfulness. They said *providing definitions of these key terms would help provide clarity on what self-compassion actually is*, and consequently increase interest in self-compassion programmes. There was also confusion about what self-compassion programmes would look like in practical terms.

“I'm kind of struggling to see what the actual programme would entail. I kind of understand the basis of it but I can't really see like the final, like how it would look like in the end.” (Female, 21 years old, Participant 10).

Most young people (80%) said that now they knew more about it, they recognised the merits of self-compassion to reduce anxiety and depression. However, they said others who were not familiar might not respond this way. Young people said *time and focus should be spent raising awareness of self-compassion and dispelling some of the misconceptions around it* (e.g., associations with self-indulgence and laziness).

“Self-compassion is maybe a bit of a new concept and might take people some time to warm up to it…” (Female, 24 years old**,** Participant 11)

Seven young people (35%) mentioned *being involved in the research process and learning more about self-compassion had raised their interest* to the point where they would actively engage with a self-compassion programme.

#### Theme 3. Self-compassion programmes are appealing but initial engagement can be difficult

A large proportion of the young people (75%) said *information about mental health concepts should be dynamic—using videos and/or animation—reasonably brief and tailored to individuals*. Young people *stressed the importance of tailoring programmes and methods of engagement to individuals or groups*, with particular consideration for family environment, culture, sexual identity and specific challenges faced. They had a range of ideas for how to engage young people with self-compassion programmes*—*for example, while some said a video would be good, others said it would be too impersonal.

Young people said consideration should be given to the *platforms used to promote mental health concepts and/or programmes*. While social media may reach a wide audience, most said they were unlikely to engage with serious content on such platforms.

“I definitely think social media is a big one. But I think you run the risk of people just scrolling past it… A lot of serious topics on social media, people don't really want to look through…” (Female, 16 years old, Participant 12).

Young people responded positively to a suggestion for a short quiz, linked from social media, to help them determine how self-compassionate they are or how much they would benefit from a self-compassion programme.

“I think (a quiz) is an acknowledgement that it's not the same for everyone, obviously we've talked a lot about that, but it can't be the same programme…it will pay attention to you and what you need.” (Female, 21 years old**,** Participant 13).

## Discussion

The purpose of this review was to synthesise the existing literature and expert opinion (young people and researchers), to determine evidence for self-compassion as an active ingredient of interventions for symptoms of anxiety and depression in youth. We believe that combining evidence from these sources provided a comprehensive and inclusive approach to answering the overarching research question about how the proposed active ingredient of self-compassion may or may not work, and why.

### What Works?

There is some evidence that interventions to increase self-compassion reduce symptoms of anxiety and depression in young people aged 14–24, when the intervention is of a sufficient length (minimum 4 sessions) and when it is a specific self-compassion intervention, as opposed to brief (e.g., 2 sessions) mindfulness training. Evidence was found to support group and individual formats, in both high school and university settings. However, these conclusions should be considered cautiously due to a number of limitations. First, there have only been a small number of controlled trials conducted to date. Second, while we judged the content of the intervention as self-compassion based on treatment manuals where possible, some were on the basis of more limited information contained in the publication. Future research should judge interventions as being self-compassion based on treatment manuals for all studies. Third of the trials conducted, the majority had small samples sizes and are likely to have been underpowered. Fourth, most studies were not treatment studies targeting elevated symptoms of anxiety and depression and were not prevention trials. Consequently, this limits generalisations regarding the efficacy of self-compassion interventions in young people. Future research on self-compassion interventions should examine mediation to determine which are the effective elements of treatment in young people. Fifth, we did not include qualitative studies in the review due to the limited number of studies, and future reviews should seek to include these in order to generalise results more broadly to young people beyond those who were consulted in this study. There is a need for more qualitative studies, particularly given the importance of increasing youth involvement in the co-creation and development of interventions for anxiety and depression. Finally, a limitation is that higher study quality was associated with smaller effect sizes when examining associations between self-compassion and anxiety. This limitation of study quality, in combination with the limited number of controlled and repeated-measure designs, indicates that the pooled effect sizes may have been slightly inflated and should therefore be interpreted relative to these limitations.

### What Does Not Work?

Brief mindfulness and loving-kindness training did not appear to work. Additionally, an important barrier to engagement identified through consultation was the belief that self-criticism helps achievement, and this should be addressed in future self-compassion interventions.

### Young People’s Recommendations

Young people were unanimous that self-compassion was relevant to their experience of anxiety and depression. Importantly, young people identified self-compassion as being opposite to self-criticism. They explained the idea of being kind to yourself and recognising that everyone struggles is hard when your usual mindset is focused on goals and striving for excellence. Young people felt more programmes were needed to teach self-compassion, and these should be tailored to individual preferences in delivery (e.g., group, individual, online) and be inclusive of diversity in gender, culture, sexuality and individual experiences. Both young people and researchers recognised that adolescence is a ‘powerful moment’ to learn about self-compassion because young people face numerous developmental challenges and stressors. Indeed, young people highlighted family pressures, exam pressures and general stress making it harder to be self-compassionate.

There are several limitations of the youth consultation we engaged in. It is important to note that the Youth Advisory Group did not report previous engagement in self-compassion interventions. We did not specifically seek young people to engage in the consultation process who had completed a self-compassion intervention, and this is a limitation as it may have been difficult for young people to share their views on an intervention of which they did not have experience. Despite this limitation, young people’s lived experience of anxiety and depression provided important and meaningful insights regarding self-compassion, for example, with some commenting on the negative connotation of words like ‘self-compassion’, which is important for researchers to consider when engaging young people in interventions. Another limitation is that we did not ask young people for details of their race or ethnicity, and the Youth Advisory Group were all from Australia. Hence the findings should be considered in the context of the views of young people in a high-income country. Future research should seek to engage young people from a broader geographical area, including low- and middle-income countries. A final limitation is that some of the interview questions asked questions such as whether the young person thinks self-compassion relates to their own, or their friends, experiences of symptoms of anxiety or depression. It is possible that because the interviews were conducted by research assistants employed in the study that young people may potentially have thought the interviewers had a vested interest in this research question, so may have been more likely to say yes to these types of questions. Hence, this potential limitation of young people potentially agreeing with the questions should be considered, and future research should seek to consult young people from another perspective, for example, with young people with lived experience of anxiety and depression leading the consultation process rather than research assistants.

### Future Research Directions and Policy Implications

Despite some debate over definition, a positive feature of the literature was self-compassion was measured consistently, with all but one study using the SCS (Neff, 2003a). This consistency in measurement strengthens conclusions regarding self-compassion, as averaging the findings across the same scale means that the studies were measuring self-compassion in the same way. As noted in previous meta-analyses (Marsh et al., 2018), higher self-compassion was related to lower symptoms of anxiety, *r* = − 0.49, 95% CI (− 0.57, − 0.42), and depression*, r* = − 0.50, 95% CI (− 0.53, − 0.47). Nevertheless, while we did not predetermine the particular definition of self-compassion that would be used in the review, the use of the SCS (Neff, 2003a) in almost all studies included may have resulted in the findings being more closely aligned with Neff’s (2003a) definition. Hence, future reviews may seek to include a broader range of measures of self-compassion which are in line with other definitions of compassion, such as Gilbert (2017).

There were few intervention studies specifically focused on building self-compassion with young people, and few controlled trials of specific self-compassion interventions (e.g., Arimistu, 2016; Bluth et al., 2016). Based on feedback from young people, future self-compassion programmes need to be available in multiple formats (online, individual, group) as there is no ‘one size fits all’ for preferred mode of delivery. Also, self-compassion programmes should be co-designed with young people and using language they find appropriate (e.g., not using the word ‘intervention’). Future research should compare self-compassion treatments with active treatments, such as Mindfulness Based Cognitive Therapy, or CBT for perfectionism which has efficacy for anxiety and depression in adolescents (e.g., Shu et al., 2019) and focuses on reducing self-criticism, and examine changes in self-compassion. Treatment trials with larger samples are needed as most were small. Trials that include young people with elevated anxiety and depression are required as well as longer follow-up periods, since only one study to date included young people with elevated depressive symptoms (Burke et al., 2020). Future research should also compare the efficacy of self-compassion interventions in adolescents compared to young adults, given studies of psychotherapy for depression have indicated greater effects in young adults than adolescents (Cuijpers et al., 2020).

Engagement with young people in the review process was critical as their views have highlighted the importance of targeting self-criticism in a manner which is seen as acceptable and does not detract from their ability to succeed and attain their goals. The findings highlight the importance of future research maximizing youth involvement in developing and tailoring interventions, with young people as co-researchers who design the interventions. Further, while the findings of our consultation with young people and researchers highlighted some important mechanisms by which they view self-compassion may influence symptoms of anxiety and depression, such as increasing self-acceptance and decreased rumination, on the basis of the results of the systematic review component, questions of how and for whom self-compassion interventions work need to be explored in future research. This is particularly important given the various definitions of self-compassion and intervention approaches in the literature, to improve understanding of reasons for the associations between self-compassion and anxiety/depression in young people.

## Conclusions

There is clear evidence that higher self-compassion is associated with lower anxiety and depression in young people. Young people recognise the importance of self-compassion in relation to anxiety and depression and in particular, noted the need for tailored treatments which are inclusive and address diversity. Further controlled trials of self-compassion intervention programmes are required to add to the emerging evidence that increasing self-compassion in young people reduces anxiety and depression.

## Supplementary Information

Below is the link to the electronic supplementary material.

Supplementary file1 (DOCX 37 kb)
