# Supplementary material for: A Review of Self-Compassion as an Active Ingredient in the Prevention and Treatment of Anxiety and Depression in Young People
Source: Adm Policy Ment Health. 2021 Sep 24;49(3):385–403. doi: 10.1007/s10488-021-01170-2 (PMC9005396; doi:10.1007/s10488-021-01170-2)
Supplement: Supplementary file 1 — Supplementary file1 (DOCX 37 kb) [file 10488_2021_1170_MOESM1_ESM.docx]

**Supplementary Table 1. Summary of findings shared with youth advisory group prior to second round of interviews.**

What does the research say?

**Do treatments for self-compassion work?**

We found 9 scientific studies looking at treatments for self-compassion.

- **Two studies** (with people aged 10-18) found that, after 6 group sessions of self-compassion treatment, young people had lower stress, anxiety and depression.
- **Four studies** with University students (aged 18-23) looked at brief (2-4 sessions) mindfulness treatments. In two of these studies young people showed lower anxiety after treatment. Some of the studies showed treatment did not change anxiety or depression, but that is probably because the treatments were so short.
- **Three studies** looked at longer treatments (7 or more sessions) in University students. Feelings of anxiety and depression reduced in one study. The other two studies found no change in anxiety or depression.

**Do changes in self-compassion help reduce anxiety and depression?**

Two studies have looked at whether mindfulness programmes for depression and anxiety work by changing participants’ levels of self-compassion.

- **One study** tested whether the changes in depression, anxiety, and stress following a school mindfulness programme were due to changes in self-compassion during the programme. The researchers divided self-compassion into two components: self-criticism and self-kindness. They found that the changes in depression and anxiety were partly due to changes in self-criticism. They were not due to changes in self-kindness.
- **The other** **study** looked at a mindfulness programme for University students. They found that changes in social anxiety and stress were due to changes in self-compassion and acceptance.

Together, these studies suggest that one way mindfulness programmes might work is by changing self-compassion, but this is more to do with reducing self-criticism than increasing self-kindness.

**Is self-compassion related to anxiety and depression?**

Thirty studies have looked at whether a young person’s level of self-compassion is associated with anxiety and depression. These studies have been conducted with young people from all around the world, aged 11-24.

- The largest study included over 3000 young people.
- All the studies had a very similar pattern of results, with self-compassion being strongly related to both anxiety and depression.
- Young people with higher self-compassion reported lower depression and anxiety. This was a strong and statistically significant finding.

Together, these studies suggest that if you have higher self-compassion you are less likely to have high levels of depression and anxiety.

**How is self-compassion experienced and what are preferences for treatment?**

Researchers have looked at experiences of self-compassion in five interview studies.

- **Two studies** looked at compassion-focussed therapy, and young people said it helped them feel connected in a group when doing the therapy and feel comfortable being themselves.
- **In another study**, young people said they liked Mindfulness Based Cognitive Therapy because it helped make them aware of their thoughts and feelings and feel calmer.
- **In one interview study**, young people said self-compassion means making their own needs a priority, having a positive outlook even during hard times, working on making positive changes to their life, avoiding self-destructive behaviour and accepting themselves.

What do the experts say?

**What do young people say about self-compassion, anxiety and depression?**

Key points from you in our first round of interviews:

- Self-compassion might help cope with self-criticism by disengaging from negative thoughts about yourself, which may boost self-worth.
- Difficult parts of self-compassion are that it can be hard to practice self-compassion under stress and it might mean you are not trying hard in your life. Some people said the term ‘self-compassion’ might have negative connotations, and researchers should be aware of using terms like this and ‘intervention’ because of their connotations.
- When asked about preferences for self-compassion treatments, some people said they would prefer face to face and group treatments, others online/app based formats, and some people said treatment should be tailored for certain people— for example taking into account cultural differences.

**What do research experts in self-compassion say about how it relates to young people?**

Key points from self-compassion experts:

- Self-compassion is useful for young people experiencing anxiety because it provides a feeling of safety, and for people experiencing depression because it helps combat negative thoughts related to self-worth.
- The idea of self-compassion can evoke fears about failure (if I stop being hard on myself, I will stop achieving/let myself go), and this can be a barrier to young people doing self-compassion treatment.
- Self-compassion works as an active ingredient in depression interventions to combat negative thoughts about self, as self-compassion targets the things that keep depression going— like self-criticism.
- One researcher talked about two aspects of self-compassion: courage, which consists of turning towards your emotions rather than away so you can become emphatic towards them; and wisdom, which involves finding strategies to deal with emotions so you can feel better.

|  |  |  |  |  |  |
| --- | --- | --- | --- | --- | --- |

**Supplementary Table 2*.* Comprehensive themes and quotes from thematic analysis of youth interviews stage 1.**

| **Themes** | **Sub-themes** | **Supporting quotes** |
| --- | --- | --- |
| *Self-compassion (SC) as the opposite of self-criticism* | SC to reduce self-criticism and disengage from negative thoughts | “Just being compassionate towards your own failure.”  “I think self-compassion can help us to sort of disengage from negative thinking.”  “If you want to judge yourself fairly then you have to be kind to yourself because you can't judge yourself fairly if you're always being harsh on yourself.”  “Positive thinking would really help, that’s what self-compassion is really is, you know, self-positive talk and stuff, knowing that you’re worth something and just things like that would definitely help.”  “If you do kind of respect yourself, you're not gonna think, Oh I’m a failure or I can't do this.” |
|  | SC means having to sacrifice your goals and achievements | “I worry that self-compassion would – if it’s too much, then I think it would prevent me from trying to strive for excellence.”  “It feels counterintuitive to try to have compassion for yourself when there’s another part of you that’s like if you just bully yourself enough maybe you’ll get us and get on with the day.”  “Maybe I guess with anxiety and everything you're very anxious all the time and you may feel that your, what you're doing is not enough.”  “In my group we’re all year 12s and half of us do ATAR so the kids that are doing ATAR, we have self-compassion for other people but not ourselves.”  “I've been told there are ways to have self-care, but A. some of them don't apply, and B. I’ll actively ignore them in order to keep overworking myself.”  “It feels like if you take the pressure off yourself, you're just gonna get worse. Like, if you feel compassionate to yourself, you're just not going to achieve the things you want to be achieving in the day.”  “Self-compassion and self-care conceptually are great but if we do them, then we lose time towards doing things that we might not end up doing anyway.”  “I can't be having that compassion because I'm clearly not good enough I'm not doing well enough... if I start being nice to myself then that's not going to get me anywhere. [SE1] Whereas, if I just try to get this thing done, or I beat myself up about it maybe something will click, and I'll do it because failure is just not an option.”  “I guess just finding that balance between being self-compassionate, but still, I guess achieving your goals.” |
|  | Stigma that SC means you are weak/lazy | “I think definitely as a teenager I think it’s a little bit stigmatised as you’re weak or it’s a little bit cringy maybe to try and do these things for yourself.” |
|  | Hard to get into the SC mindset, particularly in stressful/high pressure moments where self-criticism is high | “I think it's tricky because when you're sort of like, in the moment, it's really hard to break out of it and think about that sort of thing.”  “It’s easy to just say just be nice to yourself kind of thing but it's kind of hard to put it in practice.”  “It's kind of difficult to do when you're in the moment, and your brain’s going a million miles a minute.”  “I think it is very like, you have to trust in yourself to really be able to sort of make any change, but then that trust is something that can be really difficult when you're in a negative spiral.”  “Sometimes people are so stuck in the current situation, and they can only think about what is happening right now in front of them, but they're not able to think about, you know, the future and the broader situation.” |
|  | Self-criticism means young people treat themselves less compassionately than they do others | “I set myself much higher standards for in terms of treating myself how I would treat other people.”  “I almost question I guess and feel a bit guilty about being kind to myself so I'd say it's a barrier and I guess it's hard to find a balance of oh am I being selfish now kind of thing.”  “It's like we have that compassion for others but we then don't have ourselves and it'd be really great if we could get over that.”  “I feel like you kind of get really good at making excuses for why you're different to other people. it's easier to just, I guess be hard on yourself and not see yourself through that compassionate lens. Whereas, it's much easier to do it I guess externally.” |
| *Young people’s understanding and awareness of self-compassion* | Limited exposure to the concept of SC and need to raise awareness | “Not that specifically...I guess, self-love and things like that, it's kind of thrown around, but never specifically self-compassion, not really sure about it.”  “I think yeah online programmes and you're kind of just getting awareness out, would be like a really good start.” |
|  | Assumption it is the same as self-care | **“**I just assume it's sort of a form of like self-love and like self-care.”  “It makes me think a little bit of like you know, hashtag self-care, kind of stuff on Instagram and all that.” |
|  | Increased SC can help reduce anxiety and depression | “If you don't have self-compassion, then you're not going to find the will to, if you are depressed or have anxiety, you're not going to find the will to get better, and you're not going to love yourself enough to be able to feel happy.”  “If you learn how to have self-compassion, and learn how to treat yourself better, it can probably help with symptoms of anxiety and depression.”  “If you have more self-compassion then you’re less likely to be anxious and depressed.” |
|  | SC dependent on family background/relationships/culture | “Some of my friends who have Asian parents are very strict on test scores, and they like freak out if they like don't get above a certain grade and they don't have any forgiveness to themselves.”  “I have a friend who has struggled with that, because at home she has less of a safe environment I guess which makes it harder for her.”  “I come from like a really strict family, I doubt that my parents would be like to me, Oh you should practice self-compassion, I think it's something you should learn in a way if you're not like exposed to that sort of content.”  “I'm from Singapore, so we're not really taught about it ever – like self-compassion or just therapy in general, there's not much awareness.” |
| *Preferences for SC programmes* | SC programmes are needed and would be helpful | “I think very applicable to say anxiety, which is around these negative thought patterns, and I think practicing that self-compassion will help you realize that you know you are in those negative kind of cycles.” |
|  | SC programmes tailored to person’s preferred format and to specific groups (e.g. Culture, LGBTIQ+) | “I think definitely having something that's a bit more applicable to each individual person, and kind of putting it into your own life rather than just having these very broad and basic strategies.”  “If there is an LGBTQI plus specific part, or even service or however this looks, it needs to really focus on that and go for the idea that you know queer people are acceptable... it really needs to be focused on celebrating us in a way that sort of says, You are worthy and, you know, this is our baseline and here you've also got this amazing support.”  “I'd say like it'd be cool to have like targeted support but at the same time, sometimes when you have targeted support, there are a lot of people that miss out either because you know they can't go because of family reasons or personal reasons or so on.” |
|  | Researchers should be aware of how certain words/behaviours come across to young people | “I found more about the world in a way so words like terms like self-compassion, self-love, mindfulness, they sort of, they feel like buzzwords to people, and in a way have negative connotations because of that.”  “Rather than being something positive people kind of roll their eyes a bit, and so don't take them seriously.”  “There’s the people who try and be really cool and connect with us and whatever and it never really works because they're always way older than us.”  “I have like barely ever heard that word (intervention) used like in real life, I've always heard it like in like American movies, things like that and they're like, all we give you like an intervention and it's really, really, really bad situation... self-care also has negative connotations.” |
|  |  |  |

Note. SC = self-compassion, LGBTIQ+ = Lesbian, Gay, Bisexual, Trans, Intersex and Queer plus.

**Supplementary Table 3. Comprehensive themes and quotes from thematic analysis of youth interviews stage 2.**

| **Themes** | **Sub-themes** | **Supporting quotes** |
| --- | --- | --- |
| *Research and expert opinions were relevant to lived experience of anxiety and depression* | Focusing on reducing self-criticism as opposed to increasing self-kindness resonates with young people | “That was really interesting, and I think that it makes sense that self-criticism would be related more or would correlate more with depression and anxiety.”  “It was saying that the self-kindness was not so effective whereas the self-criticism was more effective. And I thought that was an interesting distinction because I've always kind of thought of it as two sides of the same coin. That was quite an interesting little thing, but I can understand why.”  “I thought that was nice because I hadn't really thought about that aspect of it, like when you say self-compassion, I kind of automatically think self-kindness. So, to think about reducing self-criticism as well was nice to have on the sheet as a different idea, and that's really interesting that it's more effective.”  “I guess self-compassion can be a kind of an ambiguous term, hearing the bit about self-criticism I guess makes it a bit easier to focus... I think it’s a bit more self-explanatory.”  “I thought it was really interesting that it's the self-criticism part that may be the active factor... I hadn't considered how the self-criticism kind of plays into that, rather than increasing self-compassion.” |
|  | Framing SC in terms of courage and wisdom makes it more appealing | “What I found interesting... that aspect of courage, because I don't really think about that often, it does take a lot of courage to face a lot of what you're feeling, without just putting it to the side.”  “Maybe that ties into people thinking of self-compassion as just self-care and, you know, having a nice bath or something. They're not motivated to learn more about it or think that it could actually be useful to them, something that does take courage and is difficult.”  “It also talked about courage as being one of the aspects of that, which I think is very relevant in at least my friends that I know because having courage means that you have the courage to have that self-compassion.”  “I would be really interested in, if some sort of treatment framed it in that way, because I find the idea really innovative and I'm willing to try something more innovative than the really traditional kind of self-compassion and self-criticism.”  “I think that for me if someone would say, you should practice self-compassion, I would be like, ah, I don't know. But if someone would relate it to wisdom and you’d be a lot wiser and more courageous, I think that would appeal to me more.” |
|  | Having research to back mental health programmes helps them stand out from the sea of “self-help” methods advertised online | “I think there's a lot of stuff out there that kind of targets young people or people struggling with mental health, like stuff on Instagram, people saying, if you pay me this much I'll help you, or if you buy this face mask, wow it's amazing that will reduce your anxiety. And I think it's good to know what actually does work in amongst all that.”  “I suppose the main thing was, if it feels like it helps, then it's helpful, but then again, there is a bunch of things out there that might be other crap, that doesn't work and it's just aimed towards making some money off of young people.”  “I think it was a part where it said self-compassion might have negative connotations and I'm not sure if that meant in terms of it being misinterpreted as like self-indulgent, but I guess it was really cool that there's research to back it up, I guess that kind of stops people from being like oh what is this thing, airy fairy kind of yeah, if people like try to criticize it, it's got some um research to back it up.” |
| *The novelty of SC means it might take time for it to garner real credibility among young people* | Clarity is needed around the definition of key terms and the practical aspects of SC programmes | “We've been talking a lot about self-care and self-kindness and self-criticism and all these different things, maybe somewhere in the document, having definitions for the key term could be useful for people who might not know.”  “Because there's so many different ideas that are brought up throughout the document, I'm kind of struggling to see what the actual programme would entail. I kind of understand the basis of it but I can't really see like the final, like how it would look like in the end.”  “I still am struggling to tell the difference actually between mindfulness and self-compassion. I'm still not really sure about that.”  “I guess the term self-compassion is maybe a bit vague and people might be a little bit sceptical or not really sure what it is and that might kind of get in the way.”  “In the first part (of the summary) it says studies had groups for self-compassion, I guess I was just wondering what those groups actually did... what would a therapy for self-compassion actually look like.” |
|  | Young people need to become more familiar with SC to be able to trust it and engage with it | “I think us being introduced to the idea now has helped. It’s a neat concept that just doesn’t get talked about, so we don’t know it’s an option a lot of the time.”  “Self-compassion is maybe a bit of a new concept and might take people some time to kind of warm up to it... so I think if people know what it is exactly and have that research to back it up, I think it'd be really awesome.” |
|  | Knowing more about SC increases likelihood of participating in a program | “One of the best ways I'm engaged in services, which isn't always possible due to time constraints, is through helping focus groups sometimes, like now I'm interested in what's going to come out of this and more likely to actually engage in it when it comes out.”  “I was just gonna say I think this whole experience has been really interesting, I've never really thought of self-compassion as a thing before... and I think that I have a whole new awareness about it so if you guys work on a programme, I would definitely be interested in that.” |
| *SC programs are appealing but initial engagement can be difficult* | While information is relevant, it is unlikely to be consumed unless there has been previous engagement | “I think the hardest thing is finding a balance between catching people's attention the way an Instagram post would... and still making it seem serious.”  “I think definitely if you make it too clinical then you're not going to get any attention whatsoever... if things are incredibly clinical it just makes me anxious of stepping in the door to whatever the program would be.”  “I think the problem is capturing the attention in the first place, we were talking about how easy the document is going to have to read... but it's how you get someone to actually click the link and go to that page to read more about it.”  “I would (read it) if something made me click on it, but I don't know what would make me click on it. I probably wouldn't, if I just saw it on Instagram or Facebook but if there was something that made me click the link to the page, I might.”  “If you put (something) up on social media, you need to get the person off social media as quickly as possible because of how mindlessly we scroll through social media. Because if I saw a long post on Twitter and Instagram and things like that, I will scroll past it, I will ignore it, I will forget about it within the next five seconds.”  “(Social media) I think is quite a difficult channel to manage when you're trying to get people to actually read something quite long or engage with something on a serious level, like maybe it would be better to go through other channels.” |
|  | Information needs to be dynamic, brief, tailored to individuals, and promoted on the right platforms | “For me, one of the biggest barriers to accessing treatment, which wasn’t really on the document, is this feeling of like being time shy and time poor. And one of the things that really makes me click on stuff online is like an article saying this is a five-minute read, this is a 10-minute read or it’s a 2 minute read.”  “I think maybe try and make that more multimodal. So, incorporating videos and things like that.”  “For formatting, don't make videos, I suppose, like as the intro because those sorts of videos and things just won't capture attention either.”  “I think the quiz is a good idea, because it's a bit fun and it's, you feel like you're getting a personal kind of, not diagnosis, but I get an answer, it's like a little BuzzFeed quiz or whatever, it's just fun to do.”  “I was literally gonna say quiz as an idea, especially for social media because its kind of ticks a lot of the boxes, I think. A quiz gets you off that site onto a new one, and if it's got a timeframe you understand it's not going to take too much time, it's got you interacting with something which kind of gives you that little dopamine boost of hey I'm doing something cool.”  “Personally, I don’t do quizzes. Just noting that it won’t work for everyone.”  “When you're scrolling on your phone you don't really tend to just stop and read something, you just continue to scroll, so something like a questionnaire where it's like really quick and you're just like, oh yeah look I'll click on this, I think that will probably be a lot more beneficial.”  “It should be tailored to certain people because everybody's got different needs and you know not everybody is just your standard person, everybody's different and really the same program is not going to work for everybody.”  “I think tailored treatment is really important, especially with culture, I think there's some cultures where perhaps they value kinship and family more, and maybe the idea of self-compassion could be seen as selfish.”  “I don't just want a cookie cutter, same thing for everybody, I really need something that appeals to me personally, because not everything's gonna work for everybody.” |
|  |  |  |

Note. SC = self-compassion.

**Supplementary Table 4*.* Making Friends with Yourself (MFY) – Self-compassion group intervention overview for adolescents**

| **Overview of Making Friends with Yourself – Mindful Self-Compassion Intervention** | |
| --- | --- |
| **Session** | **Description** |
| Session 1 | **Discovering mindful self-compassion**   - Introduction to concepts of mindfulness and self-compassion - Safety measures for class established - Informal and formal practices introduced |
| Session 2 | **Paying attention on purpose**   - Concept of mindfulness, wandering mind, and default mode network discussed - Mindful eating, soles of the feet and body scan practices are presented |
| Session 3 | **Introduction to loving-kindness**   - Loving-kindness is defined and loving-kindness practice is introduced - Participants create their own loving-kindness phrases - Adolescent brain development is discussed |
| Session 4 | **Developing self-compassion**   - Exercise encourages teens to turn from the Inner Critic toward the Compassionate Voice - Music meditation is introduced |
| Session 5 | **Differentiating between self-compassion and self-esteem**   - Difference between these two concepts is elucidated - Perils of social comparison is discussed - Common Humanity is illustrated through a ‘crossing the line’ exercise |
| Session 6 | **Living deeply**   - Core values exercise clarifies values - Giving and receiving meditation is introduced |
| Session 7 | **Managing difficult emotions**   - Soften, soothe, allow practice is introduced - Tools to contend with anger and unmet needs are practiced - Two developing systems of the adolescent brain are explained |
| Session 8 | **Embracing your life with gratitude**   - Meditation – Compassionate friend - Gratitude and self-appreciation practices are presented - Wrap-up of course takes place via writing a letter to oneself, which is mailed to participants a month later |

Note. A 6 session version of the MFY programme was evaluated in: Bluth, K., Gaylord, S.A., Campo, R.A., Mullarkey, M.C., & Hobbs, L. (2016). Making friends with yourself: A mixed methods pilot study of a mindful self-compassion program for adolescents. *Mindfulness, 7*, 479-492 and Bluth, K., Roberson, P.N.E., & Gaylord, S.A. (2015). A pilot study of a mindfulness intervention for adolescents and the potential role of self-compassion in reducing stress. *Explore, 11*, 292-295.

**Supplementary Table 5. Brief Self-Compassion Training vs Attentional control**

| **Overview of Brief Self-Compassion Training** | |
| --- | --- |
| **Session** | **Description** |
| 1 | **Randomisation into groups and listening to group-specific meditation recordings once a day for three days**   - Self-compassion recording – “*may I be happy….may I be at ease*..” with recording content drawn from Neff’s conceptualisation of self-compassion. Women were asked to repeat each phrase silently with intention and self-kindness - Attention control – Recordings were from a psychological textbook |
| 2 | **Trier Social Stress Test**   - Listen to five minutes of group-specific recordings with the instructions “The rest of the study will be challenging. To help you prepare for the challenge, we invite you to listen to a recording similar to the ones you listened to at home” - Trier Social Stress Test as per Kirschbaum et al. (2008) |

Note. This brief self-compassion training was tested in Arch, J.J., Warren Brown, K., Dean, D.J., Landy, L.N., Brown, K., & Laudenslager, M.L. (2014). Self-compassion training modulates alpha-amylase, heart rate variability and subjective responses to social evaluative threat in women. *Psychoneuroendocrinology, 42*, 49-58. doi: 10.1016/j.psyneuren.2013.12.018.

**Supplementary Table 6. Enhancing Self-Compassion Program**

| **Overview of Enhancing Self-compassion Program** | |
| --- | --- |
| **Session** | **Description** |
| **1** | **Introduction and loving-kindness meditation (LKM) practice**   - Psychoeducation - Practice Loving Kindness Meditation – loved one, themselves, a stranger, a difficult person and all humans - Practice LKM for 10 minutes a day until next session |
| **2** | **Mindfulness practice**   - Review last sessions materials - Instructions on mindfulness skills (e.g. Mindful breathing) - Practice LKM and mindfulness for at least 10 minutes a day until next session |
| **3** | **Compassionate imagery skills**   - Review last sessions materials - Explore and create an image of an ‘ideal’ compassionate nurturer - Practice LKM, mindfulness and compassionate imagery for at least 10 minutes a day |
| **4** | **Compassionate ideal self**   - Review of last sessions materials - Imagining the compassionate ideal self - Practice skills learned from this and all prior sessions for 10 minutes a day |
| **5** | **Compassionate letter writing**   - Review of last sessions materials - Place themselves in the role of their ideal compassionate self and write a letter to themselves - Practice skills learned from this and all prior sessions for 10 minutes a day |
| **6** | **Three-Chair work**   - Review of last sessions materials - Three-chair work dialogue - Practice skills learned from this and all prior sessions for 10 minutes a day |
| **7** | **Compassionate behaviour**   - Review of last sessions materials - Practice compassionate behaviour in reference to real life situation (e.g. threatening event) - Discussion about experiences, review progress, and future issues |

Note. Enhancing self-compassion program was evaluated by Arimitsu, K. (2016). The effects of a program to enhance self-compassion in Japanese individuals: A randomised controlled trial. *The Journal of Positive Psychology*, *11*, 559-571. doi: 10.1080/17439760.2016.1152593

**Supplementary Table 7. Mindfulness and self-compassion sessions (Resilience training)**

| **Overview of Resilience training workshop** | |
| --- | --- |
| **Session** | **Description** |
| **1** | **Instructions to workshop and mindfulness skills**   - Discussions about purpose of workshop and resilience - Introduction to mindfulness skills - Explore possible contributions to barriers to being mindful - Engage in mindfulness skills exercises (e.g. mindful eating) - Relating mindfulness to resilience - Home practice |
| **2** | **Self-compassion skills**   - Review previous sessions and homework - Introduction of self-compassion skills - Explore possible contributions to barriers to being self-compassionate - Engage in self-compassion writing exercise - Relating self-compassion to resilience - Homework practice of self-compassion and mindfulness exercises |
| **3** | **Mindfulness of relationships**   - Review of past sessions and homework - Introduction to mentalisation skills - Explore barriers to mentalisation - Engage in mentalisation exercise exploring alternative beliefs - Relating mentalisation to resilience - Homework practices of alternative beliefs exercises |
| **4** | **Inner strength**   - Review of past sessions and homework - Engage in experiential exercise that practice mentalisation skills and alternative beliefs - Review and consolidate resilience, mindfulness, self-compassion and mentalisation - Engage in self-compassion letter writing - Discussion on how workshop skills can be used in real life |

**Note.** Resilience workshop was evaluated by Burke, A.S., Shapero, B.G., Pelletier-Baldelli, A., Deng, W.Y., Nyer, M.B., Leathem, L., Namey, L., Landa, C., Cather, C., & Holt, D.J. (2020). Rationale, methods, feasibility and preliminary outcomes of a transdiagnostic prevention program for at-risk college students. *Frontiers in Psychiatry*. doi:10.3389/fpsyt.2019.01030
